# A simple and rapid identification method for newly emerged porcine *Deltacoronavirus* with loop-mediated isothermal amplification

**DOI:** 10.1186/s40659-017-0135-6

**Published:** 2017-09-21

**Authors:** Fanfan Zhang, Yu Ye, Deping Song, Nannan Guo, Qi Peng, Anqi Li, Xingrong Zhou, Yanjun Chen, Min Zhang, Dongyan Huang, Yuxin Tang

**Affiliations:** Department of Veterinary Preventive Medicine, College of Animal Science and Technology, Jiangxi Agricultural University, Nanchang, Jiangxi China

**Keywords:** Porcine *Deltacoronavirus* (PDCoV), RT-LAMP, Rapid diagnosis

## Abstract

**Background:**

Porcine *Deltacoronavirus* (PDCoV) is a newly emerged enteropathogenic coronavirus that causes diarrhea and mortality in neonatal piglets. PDCoV has spread to many countries around the world, leading to significant economic losses in the pork industry. Therefore, a rapid and sensitive method for detection of PDCoV in clinical samples is urgently needed.

**Results:**

In this study, we developed a single-tube one-step reverse transcription loop-mediated isothermal amplification (RT-LAMP) assay specific for nucleocapsid gene to diagnose and monitor PDCoV infections. The detection limit of RT-LAMP assay was 1 × 10^1^ copies of PDCoV, which was approximately 100-fold more sensitive than gel-based one-step reverse transcription polymerase chain reaction (RT-PCR). This assay could specifically amplify PDCoV and had no cross amplification with porcine epidemic diarrhea virus (PEDV), transmissible gastroenteritis virus (TGEV), porcine kobuvirus (PKoV), porcine astrovirus (PAstV), porcine reproductive and respiratory syndrome virus (PRRSV), classic swine fever virus (CSFV), and porcine circovirus type 2 (PCV2). By screening a panel of clinical specimens (N = 192), this method presented a similar sensitivity with nested RT-PCR and was 1–2 log more sensitive than conventional RT-PCR in detection of PDCoV.

**Conclusions:**

The RT-LAMP assay established in this study is a potentially valuable tool, especially in low-resource laboratories and filed settings, for a rapid diagnosis, surveillance, and molecular epidemiology investigation of PDCoV infections. To the best of our knowledge, this is the first work for detection of newly emerged PDCoV with LAMP technology.

**Electronic supplementary material:**

The online version of this article (doi:10.1186/s40659-017-0135-6) contains supplementary material, which is available to authorized users.

## Background

Porcine *Deltacoronavirus* (PDCoV) is a member of the genus *Deltacoronavirus* in the family *Coronaviridae* [1, 2]. PDCoV was first identified in Hong Kong in 2012, and then isolated in the United States [2, 3]. Afterwards, PDCoV was reported in Korea, China, and Thailand [4–6]. PDCoV causes an acute, highly contagious, and devastating enteric disease that is characterized by severe diarrhea, vomiting, dehydration, and a high number of deaths in nursing piglets [3–7]. Clinical symptoms of infected swine are indistinguishable from those caused by porcine epidemic diarrhea virus (PEDV) and transmissible gastroenteritis virus (TGEV) [3, 8, 9]. Experimental studies on gnotobiotic and conventional piglets showed that isolated PDCoVs caused similar clinical signs to the disease of field infections, from mild to severe diarrhea and intestinal lesions [3]. Molecular surveillance on diarrheal samples of swine from USA indicated a 30% infection rate of PDCoV, and a similar frequency of PDCoV (31.33%) in China was also detected in fecal and intestinal samples of diarrheic pigs [6]. Therefore, a simple, rapid, and highly sensitive diagnostic method for detection of PDCoV is urgently needed for the prevention and control of the virus infections and spread.

Currently, available methods for detection of PDCoVs include conventional reverse transcription—polymerase chain reaction (RT-PCR), nested RT-PCR, real-time RT-PCR, and ELISA [6, 10–13]. However, these techniques have some shortcomings, such as higher requirements for equipment, high cost, extended detection period, and/or low sensitivity [14]. Reverse transcription loop-mediated isothermal amplification (RT-LAMP) provides a potential effective tool for rapid and accurate identification of viral pathogens, which amplifies nucleic acids under isothermal conditions with high sensitivity and specificity. This novel gene detection technique is cost-effective and time-saving, and only requires a constant temperature water bath. RT-LAMP has been widely used in clinical diagnosis for detection of the presence of several important viral pathogens, including PEDV, TGEV, classic swine fever virus (CSFV), and H10N8 subtype of influenza A virus [14–17]. The improved LAMP assays, such as visual detection of amplified products by adding SYBR Green I or hydroxynaphthol blue (HNB), have made it easier to apply in primary clinical settings or for field use. In the present work, we developed and evaluated a specific and sensitive RT-LAMP assay for visual detection of PDCoV, which might be a good tool for the diagnosis of PDCoV in field samples.

## Results

### Optimization of RT-LAMP assay for detection of PDCoV

**Fig. 1:**
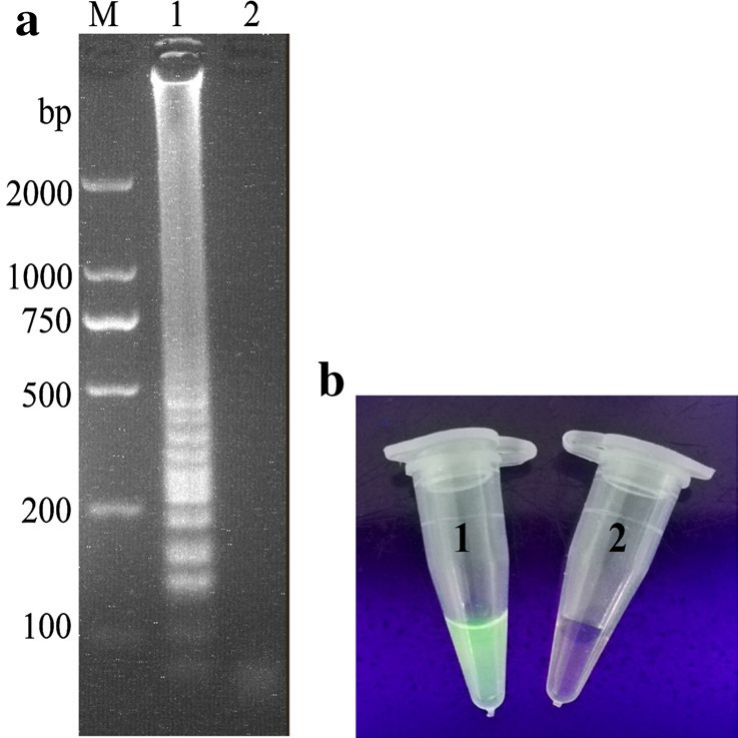
LAMP products detected by electrophoresis analysis (**a**): lane 1, positive control, lane 2, negative control, M, DL2000 DNA marker; and by visual observation with addition of SYBR green I dye (**b**): tube 1, positive control, and tube 2, negative control

The RT-LAMP assay for detection of PDCoV was successfully developed. The optimized parameters of the reaction system of the RT-LAMP were as follows: 6 mM MgSO_4_, 1.4 mM dNTPs, and 1.6 µM each of PDCoV-specific FIP and BIP primes with an incubation condition at 63 °C for 70 min in a water bath. The amplified products were electrophoresed on 2.0% agarose gel with addition of EB under UV light (Fig. 1a). The RT-LAMP products were also visually detected by adding SYBR Green I dye (Fig. 1b).

### Sensitivity of RT-LAMP with conventional RT-PCR and nested RT-PCR

**Fig. 2:**
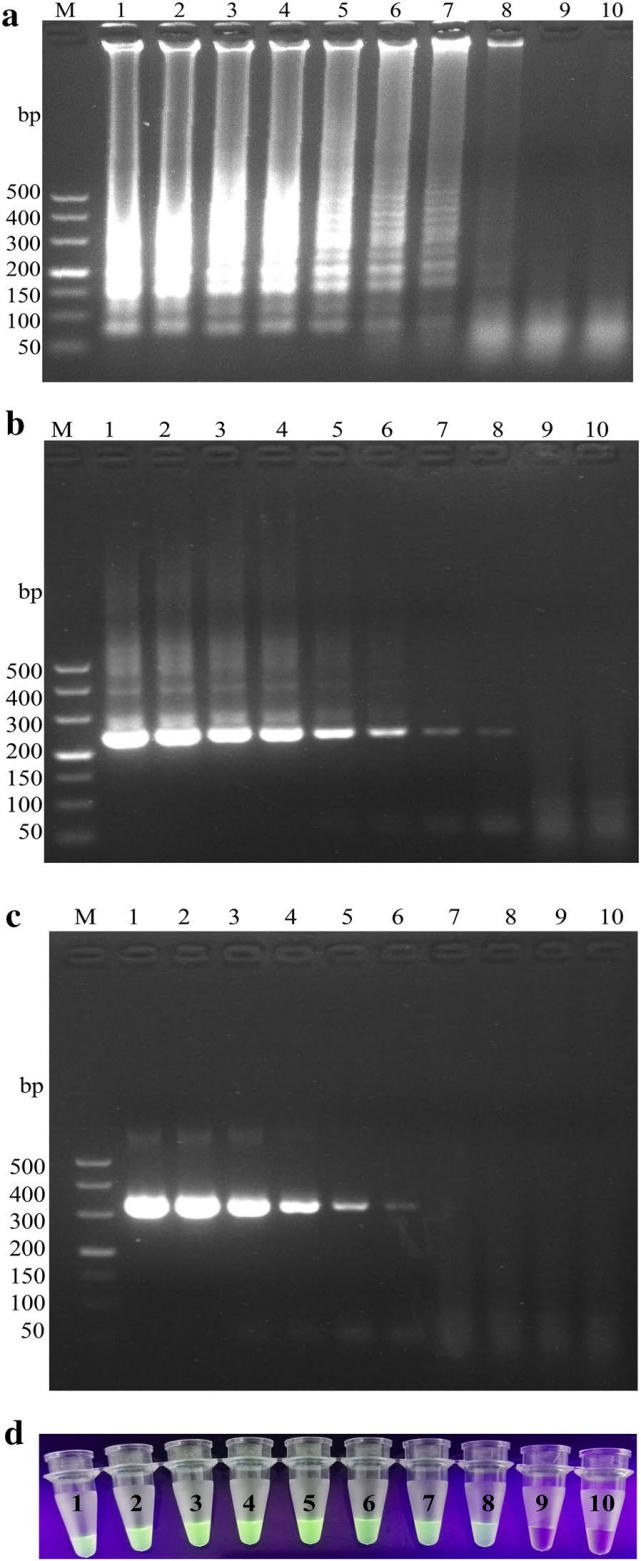
Comparison of the sensitivity of the RT-LAMP, nested RT-PCR and conventional RT-PCR for PDCoV detection by agarose gel electrophoresis analysis (**a** RT-LAMP; **b** Nested RT-PCR; and **c** conventional RT-PCR) and by visual observation (**d**). M, DL500 DNA marker; lane 1–10: a serial of diluents of the recombinant plasmid containing partial PDCoV N gene insert, i.e., 1.0 × 10^8^, 1.0 × 10^7^, 1.0 × 10^6^, 1.0 × 10^5^, 1.0 × 10^4^, 1.0 × 10^3^, 1.0 × 10^2^, 1.0 × 10^1^, 1.0 × 10^0^ copies, and negative control

The kinetic analysis of the turbidity showed that the detection limit of RT-LAMP was 1 × 10^1^ copies, which was 100 times higher when compared with conventional RT-PCR (Fig. 2a, c). As illustrated by Fig. 2b, d, the RT-LAMP assay established in this study presented a similar sensitivity with nested RT-PCR. There were no significant differences in context of the sensitivity between electrophoresis analysis and visual observation of SYBR Green I staining.

### Specificity of RT-LAMP

**Fig. 3:**
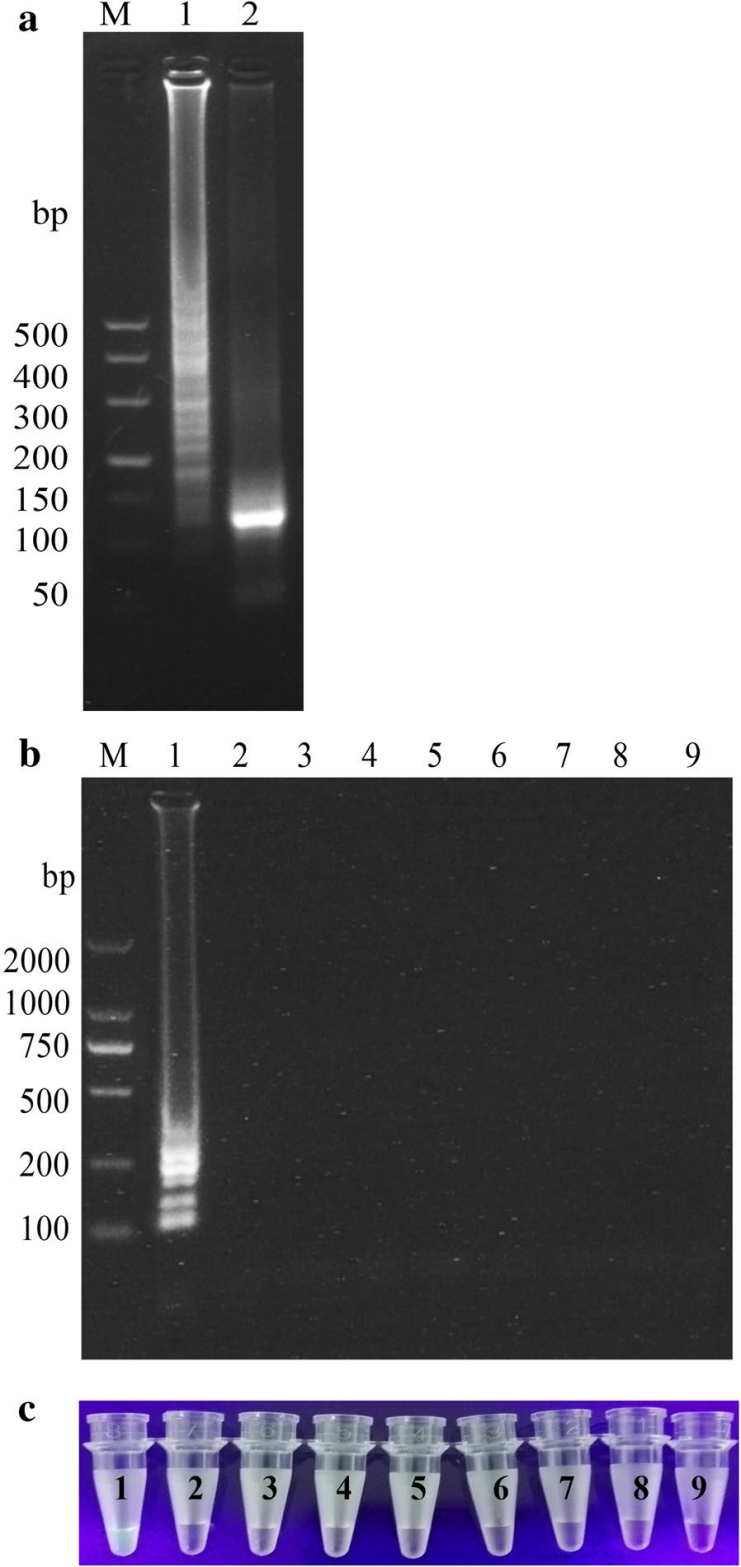
**a** Specificity evaluation of the RT-LAMP assay determined by digestion with restriction enzyme *Sml*I (**a**); lane 1, positive control, lane 2, LAMP products from lane 1 after digestion with *Sml*I; M, DL500 DNA marker; specificity of the RT-LAMP assay determined by electrophoresis analysis (**b**) and SYBR Green I staining (**c**); lane 1, PDCoV; lane 2–9, PEDV, TGEV, PKoV, PAstV, PRRSV, CSFV, PCV2, and negative control

In order to ensure the accuracy of the assay, the positive controls used in this study were initially tested for the presence of PDCoV by a conventional RT-PCR established in our laboratory. These results were further confirmed via sequencing and virus isolation methodologies. The results of specificity determination of the RT-LAMP assay (Fig. 3a, b) demonstrated that only PDCoV as the template could be amplified, and no RT-LAMP amplified products were observed for other reference porcine viral pathogens. Additionally, the RT-LAMP products were confirmed by a digestion analysis with restriction enzyme *Sml*I (Fig. 3a), and the results of enzyme digestion analysis were consistent with that from the PDCoV positive control, indicating that the RT-LAMP assay developed was specific for detection of PDCoV.

### Clinical sample detection

All the samples (N = 192) were examined for the presence of PDCoV by conventional RT-PCR, nested RT-PCR, and RT-LAMP. As indicated in Table 1, the positive rates of conventional RT-PCR, nested RT-PCR, and RT-LAMP were 32.8% (63/192), 37.5% (72/192), and 38.0% (73/192), respectively. It is indicated that our data are consistent with PDCoV prevalence in Chinese swine [6]. The detailed results of detection (case by case) are listed in Additional file 1: Table S1. The results demonstrated the RT-LAMP assay established was the most sensitive method among these three assays for detection of PDCoV in field samples.

**Table 1:** Detection results of PDCoV in clinical samples by conventional RT-PCR, nested RT-PCR and RT-LAMP

Specimen type	Total number of samples	Positive rate of PDCoV		
		Conventional RT-PCR	Nested RT-PCR	RT-LAMP
Feces	53	26.4% (14/53)	32.1% (17/53)	34.0% (18/53)
Intestinal contents	139	35.3% (49/139)	39.6% (55/139)	39.6% (55/139)
Total	192	32.8% (63/192)	37.5% (72/192)	38.0% (73/192)

## Discussion

PDCoV was first described as a newly emerged coronavirus in swine from rectal swabs in 2012 by Woo [2]. Since then, PDCoV infections have been reported in America, Europe, and Asia, and caused substantial economic losses [4–7, 18, 19]. As a result, it is urgently needed to develop an easy, rapid and highly sensitive diagnostic method for detection of PDCoV. The current methods for the diagnosis of PDCoV include conventional RT-PCR, nested RT-PCR, real-time RT-PCR, and ELISA. However, these methods are inappropriate for detection of PDCoV in the field settings and resource-poor laboratories, due to lack of sophisticated instruments.

In this study, a RT-LAMP assay was developed and evaluated for PDCoV detection. A set of primers was designed against the conserved coding regions of the N gene of PDCoV and the reaction conditions were optimized. The RT-LAMP assay was able to detect PDCoV with a detection limit of 10 copies, which was 100-fold more sensitive than conventional RT-PCR. Furthermore, the RT-LAMP assay only needs a water bath for 70 min incubation to accomplish efficient amplification, which was much fast and simple when compared with conventional RT-PCR and nested RT-PCR.

It is obvious that the RT-LAMP has some advantages over the conventional PCR-based tests and serological tests. The selected set of four primes specific to the N gene of PDCoV dramatically enhanced the specificity of RT-LAMP assay in contrast of the traditional diagnostic methods [4–7, 18, 19]. The fact that the RT-LAMP assay could not detect several reference swine viruses (PEDV, TGEV, PKoV, PAsTV, PRRSV, CSFV, and PCV2) demonstrated that this method established was highly specific. In addition, the results of sensitivity/detection limit comparisons indicated that the assay was the most sensitive among the tests employed for the evaluation of the assay in this study. Moreover, the RT-LAMP was a one-step assay in which the reverse transcription and LAMP reaction were combined, which further simplified the procedure and shortened the time of the reaction of the assay. The RT-LAMP was simple and user-friendly, and only required a water bath with a constant temperature feature or a traditional heat block to perform. To evaluate the practicability of RT-LAMP in the field, 192 clinical diarrhea samples of piglets were examined. The results showed that the RT-LAMP had a similar sensitivity with nested RT-PCR and was much sensitive than conventional RT-PCR in detection of PDCoV. These data further suggested that PDCoV was a leaved out pathogen related to swine diarrhea in China, and might cause the high mortality in diarrheal piglets.

## Conclusions

This one step RT-LAMP established in this study will provide an effective technique tool for the rapid diagnosis, surveillance, and the investigation of molecular epidemiology of PDCoV.

## Methods

### Sample collection

All samples, including feces and intestinal contents, were collected from suckling piglets of 1–2 weeks old on 35 pig farms with acute diarrhea outbreaks in Jiangxi, China during 2014–2015, and were tested for PDCoV, PEDV, TGEV, CSFV, porcine kobuvirus (PKoV), porcine astrovirus (PAstV), porcine reproductive and respiratory syndrome virus (PRRSV), and porcine circovirus type 2 (PCV2) by RT-PCR or PCR. Corresponding positive controls of the viruses used in the study are preserved in our labs.

### RNA/DNA extraction

The total RNAs of the samples were extracted by using the RNAiso Plus (Takara, Japan) and the genomic DNAs of PCV2 were extracted with the DNAiso reagent (Takara, Japan) according to the manufacturer’s instructions. The extracted RNAs/DNAs were dissolved in 30 μL of nuclease-free water. RNA samples were stored at −80 °C and DNA samples were stored at −20 °C until use.

### Targeted region selection and LAMP primers design

To identify the conserved regions, the complete genome sequences of PDCoV currently available in GenBank, including KR131621, KX083667, KP757891, K981395, KJ567050, KT021234, KT266822, and KY065120, were analyzed by the Vector NTI^®^ Advance 10 program (Invitrogen Corp, USA). The conserved sequence fragment of the nucleocapsid (N) gene of PDCoV was selected as the targeted region, and used to design the PDCoV LAMP primers by the Primer Explorer V4 software (http://primerexplorer.jp/e/). All the primers designed were synthesized by Sangon Biotech Co., Ltd. (Shanghai, China) with PAGE purification. The detailed information of the primers for the RT-LAMP is listed in Table 2.

**Table 2:** The primers for detection of PDCoV used in this study

Test	Primer ID	Type	Length (bp)	Sequence (5′–3′)
RT-LAMP	PDCoV-F3	Forward outer	18	CGGCTCTGCAGACACTGA
	PDCoV-B3	Reverse outer	19	CCGTATTGAGCGCATCCTT
	PDCoV-FIP	Forward inner	42	GCATTTCCTGAACACCAGGCACGAAGACGGGTATGGCTGATC
	PDCoV-BIP	Reverse inner	41	CTGGCCACCTTGAGAGCAACTAGAACCCTCCTTGACTGTGA
RT-PCR	PDCoV-NF	Forward	22	CCAAACGCAACCCCAACAATCC
	PDCoV-NR	Reverse	22	CTTCTCAGTGTCTGCAGAGCCG
Nested RT-PCR	PDCoV-OF	Forward outer	20	TGCTACCTCTCCGATTCCCA
	PDCoV-OR	Reverse outer	20	ATCCTGTTTGTCTGCTGGCA
	PDCoV-IF	Forward inner	22	GACACTGAGAAGACGGGTATGG
	PDCoV-IR	Reverse inner	22	TAGTTGGTTTGGTAGGTGGCTC
Standard control	PDCoV-NWF	Forward	21	ATGGCCGCACCAGTAGTCCCT
	PDCoV-NWR	Reverse	20	CTACGCTGCTGATTCCTGCT

### The construction of standards

In order to construct the standards of PDCoV N gene, the targeted RNA of PDCoV was reversely transcribed into single-stranded cDNA by a random primer and then amplified by PCR using forward primer PDCoV-NWF and reverse primer PDCoV-NWR (Table 2). The reaction conditions were as follows: denaturation at 95 °C for 5 min, 38 cycles of 94 °C for 30 s, 53 °C for 30 s, 72 °C for 2 min, and a final extension at 72 °C for 7 min. The amplified products were purified by E.Z.N.A™ Gel Extraction Kit (Omega, USA) and subsequently cloned into *E. coli* JM109 using the pGEM-T easy vector (Promega, USA). The recombinant plasmid was extracted using TIANprep Mini Plasmid Kit (TIANGEN, China) following the protocol of the manufacturer. The concentration and quality of the plasmid DNA was determined by NanoDrop 2000 spectrophotometer (Thermo scientific, USA), which was then used as the standards for the quantitative analysis.

### RT-LAMP

The RT-LAMP reaction was carried out in a final volume of 25 μL. To optimize the reaction parameters, reactions containing different concentrations of MgSO_4_ (at 2, 4, 6, 8 mM, and 10 m, Sigma, USA), dNTPs mix (at 1.0, 1.2, 1.4, and 1.6 mM, Promega, USA), each of inner primer FIP and BIP (at 0.6, 0.8, 1.0, 1.2, 1.4, and 1.6 µM), each of outer primer F3 and B3 (0.2 µM), Bst DNA polymerase (8 U, New England Biolabs, USA), AMV reverse transcriptase (2 U, Takara, Japan), betaine (0.8 M, Sigma, USA), 2 μL of RNA template per reaction were tested. Furthermore, the temperature of the RT-LAMP reaction was determined by incubating the reaction systems at 60, 61, 62, 63, and 64 °C for 70 min in a water bath, respectively, and then the reactions were terminated by heating up at 80 °C for 10 min. The RT-LAMP products were electrophoresed on 2.0% agarose gel in 1× TAE buffer, or directly visualized by adding 1× SYBR green I in the reaction system for diction by a color change. In addition, the RT-LAMP products were identified by the digestion of *Sml*I (New England Biolabs, USA).

### Conventional RT-PCR and nested RT-PCR

To identify PDCoV in diarrheal samples of piglets, a N-gene-based conventional RT-PCR assay previously established in our lab was employed [10]. The first-strand cDNA was synthesized with reverse primers PDCoV-NR, followed by PCR with primer pairs of PDCoV-NF and PDCoV-NR under the following conditions: denaturation at 94 °C for 5 min, 38 cycles of 94 °C 30 s, 54 °C 30 s, 72 °C 40 s, and consequently with a final extension at 72 °C for 10 min. Expected PCR products of 329 bp in size were purified, cloned and sequenced. The nested RT-PCR method for detection of PDCoV was performed as reported previously [6]. Finally, the amplicons were subjected to electrophoresis on 2% agarose gel, and the target bands were visualized under UV light by staining with ethidium bromide.

### Determination of the sensitivity and specificity of the RT-LAMP assay

To determine the sensitivity of the RT-LAMP assay, the constructed recombinant standards with known concentration were made tenfold serial dilutions (from 1 × 10^8^ to 1 × 10^0^ copies), and served as the templates for conventional RT-PCR, nested RT-PCR and the RT-LAMP assay. To evaluate the specificity of the RT-LAMP assay, RNAs/DNAs of several important pathogenic viral agents of pigs, including PEDV, TGEV, PKoV, PAsTV, PRRSV, CSFV, and PCV2, were used in the RT-LAMP assay under optimized reaction conditions. The standards of PDCoV and blank template were served as the positive and negative controls, respectively. All reactions were carried out in triplicates.

### Detection of PDCoV in clinical samples by conventional RT-PCR, nested RT-PCR, and RT-LAMP

A total of 192 fecal/intestinal clinical samples of piglets with severe watery diarrhea were collected from 35 pig farms in Jiangxi, China during 2014 and 2015. All samples were then selected to detect the presence of PDCoV by conventional RT-PCR, nested RT-PCR, and RT-LAMP. The samples were resuspended in saline, subsequently vortexed and then centrifuged at 8000×*g* for 5 min at 4 °C. The supernatants were harvested and stored at −80 °C until use. Viral RNA was extracted and used as a template for conventional RT-PCR, nested RT-PCR, and RT-LAMP according to aforementioned methods.

## Additional file

**Additional file 1: Table S1.** Positive rate of PDCoV determined by three PCR-based assays.
